# Letter to the Editor Concerning “Does Erector Spinae Plane Block Decrease Analgesia Requirements after Minimal-Invasive Posterior Transpedicular Stabilization in Patients with Vertebral Body Fracture?”

**DOI:** 10.1177/21925682231166903

**Published:** 2023-03-27

**Authors:** Pravesh S. Gadjradj, Sean Cofano, Fabian Sommer, Mateusz Bielecki, Rodrigo Navarro-Ramirez, Judith de Rooij

**Affiliations:** 1 Department of Neurological Surgery, New York-Presbyterian Och Hospital/Weill Cornell Medical Center, New York, NY, USA; 2 Department of Anesthesiology, Lenox Hill Hospital, New York, NY, USA; 3 Department of Neurological Surgery, Mayo Clinic Jacksonville, FL, USA; 4 Department of Anesthesiology, 6993 Erasmus MC, University Medical Center Rotterdam, the Netherlands

## To the Editor

Its with great interest that we read the paper by Holas et al. investigating the effectiveness of an Erector Spinae Plane Block (ESPB) on postoperative opioid consumption and pain as measured on the visual analogue scale (VAS) in a randomized, placebo controlled, double blind trial in patients undergoing a minimally invasive posterior stabilization.^1^ The authors included a total of 60 patients and concluded that the ESPB reduced the opioid consumption during PACU stay by 38% and reduced the VAS. Even though we appreciate the paper by Holas et al and its potential role in combatting the opioid crisis, some methodological issues need to be discussed or clarified.^2^

First is the absent registration of the study. Publication of trials can be both time consuming and costly, as well as be a burden to patients. Therefore, meticulously written protocols, with detailed formulation of in and exclusion criteria, calculation of the sample size and detailed description of the randomization blocks and analysis method, are warranted. In doing so, researchers can both increase the validity of their research as increase the reproducibility of the trial. Therefore, most journals nowadays, including the Global Spine Journal, ask authors to provide the clinical trial registry number and CONSORT-reporting guidelines for transparency reasons.^3,4^

Second is the reporting and consequent interpretation in the current study. The authors briefly mention a CONSORT Flow Diagram but didn’t include this in their publication. Furthermore, using the CONSORT-reporting guidelines would also improve the reporting as it for instance (1) asks authors to report who was exactly blinded during the trial (the anesthesiologist, the patient, the researcher and/or the surgeon); and (2) recommends against statistical testing for baseline differences in randomized studies. Other issues related to this is the question how the VAS was measured. Usually, the VAS is a score ranging from 0 to 10 (or 100) on which the patient places an x on a line based on how much pain they are experiencing. Therefore, it is a bit confusing to understand how the VAS reported by the authors can be so detailed measured that there are 3 numbers behind the period which may also affect the statistical testing. Nevertheless, the reported difference in VAS is very well below commonly accepted minimally clinically important difference thresholds (usually ranging from 10 to 25%).^5^

Thirdly and finally we therefore disagree with the authors conclusions. As the primary objective of the study was aimed at measuring differences in opioid consumption and VAS during the first 48 hours after surgery, a more valid conclusion would be to state that: “There were no statistical differences in both morphine consumption and VAS for pain during the first 48 hours of surgery. Some statistically significant differences were found between both patients groups at some measurements, but these were all not clinically relevant.”

Again, we would like to congratulate the authors with their work and look forward to their reply.
